# Pre-Medical Students’ View Points on Integrated Poster Presentations as a Tool for Learning Medical Science

**DOI:** 10.21315/mjms2018.25.6.12

**Published:** 2018-12-28

**Authors:** Rashmirekha Sahoo, Shahzaib Rehan, Soumendra Sahoo

**Affiliations:** 1 Foundation in Science, Melaka Manipal Medical College, Manipal Academy of Higher Education (MAHE), Bukit Baru, 75150 Melaka, Malaysia; 2 University Hospital of Wales, Cardiff, United Kingdom of Great Britain and Northern Ireland; 3 MEU, Faculty of Medicine, Melaka Manipal Medical College, Manipal Academy of Higher Education (MAHE), Bukit Baru, 75150 Melaka, Malaysia

**Keywords:** critical thinking, collaborative learning, poster presentation, integration

## Abstract

**Background:**

A poster presentation is an experiential learning activity that stimulates curiosity and interest among students. Moreover, it encourages exploration and integration of concepts and provides students with a novel way to demonstrate their understanding of scientific principles. This pilot projects aimed to analyse views of participants on the academic benefits and learning of medical sciences via poster presentations.

**Methods:**

This cross-sectional study used the sequential exploratory type of mixed methods design in which quantitative data analysis was performed via survey-based questionnaires and qualitative study. For this purpose, we performed a thematic analysis of semi-structured interview questions that were administered to all participants using the self-interview technique.

**Results:**

A majority of students were of the opinion that the process of making poster preparation acted as an opportunity to promote deep learning. Moreover, a majority expressed that making these presentations required teamwork, which gave them an insight into collaborative learning.

**Conclusion:**

Our study revealed that poster presentations, when used effectively as an assignment, can facilitate a learner’s critical and reflective thinking and promoting active learning. Previous generic guidelines for making posters were found to be an important step that led to a systematic scientific approach amongst learners as well as for integrating basic science and medical knowledge.

## Introduction

A poster presentation is a dynamic and interactive experiential learning activity that stimulates curiosity and interest amongst students. Students find poster presentations enjoyable, rewarding and are familiar and comfortable with them (1, 2). Collaborative work between students encourages development of teamwork and communication skills. Previous studies have suggested that deeper learning is promoted via hands-on tasks for students who create posters (3). Both creative skills and research skills benefit through the active process of poster construction (4). The medium of posters encourages a succinct presentation of information, exploration and integration of concepts, and helps in the transfer of knowledge too. Moreover, it provides students with a novel way to demonstrate their understanding of scientific principles and encourages reflection too (5).

In higher education, specifically in medical education, educators continuously stress on the importance of applying critical thinking to all facets of academic study; be it to select information, to read, to write, to speak or even to listen. Critically reading and evaluating information are seen by some as a very important skill that has to be developed as they have wide applicability in other aspects of cognition. In healthcare-related courses, critical thinking remains a vital skill to hone as it can aid optimal performance at future workplace scenarios. This approach enables learners to make an evaluation of current knowledge along with fostering the creation of new knowledge. However, it is not surprising to note that the majority of health science courses acknowledge the magnitude and importance for learners to establish critical thinking skills. To deliver effective health care whilst maintaining safety for healthcare seekers, healthcare workers are required to develop effective clinical reasoning and acumen (6). For similar reasons, critical thinking is considered to be important for practicing medicine and health sciences, in health science research, and in the philosophy of medicine itself. Critical thinking can provide broader perspectives, creative solutions, multiple pathways and scope for more self-regulation (7). Studies have shown that critical thinking can be taught; however, it should be performed in such a way that learners are actively engaged with teachers who transform themselves as facilitators (8).

The Foundation in Science (Fis) programme, at Melaka-Manipal Medical College (MMMC) in Malaysia, is a precursor course for aspiring medical or dental students. During the first semester of the Fis programme, a poster presentation competition is conducted in small groups. An emphasis is placed on the retrieval of scientific information with respect to certain medical technologies, in which the principles of basic science have been incorporated.

This pilot project was conducted to analyse the views of Fis students about the merits of using poster presentations to educate in academia.

## Methods

This was a cross-sectional study that used a sequential exploratory model of mixed methods design, in which the participants were from Foundation in Science programme. This is a one-year programme of three semesters. Some basic aspects of medical science like anatomy, physiology, pharmacology, forensic science and oral biology are included with traditional subjects such as physics, chemistry, biology and mathematics. During the second semester, one of the group tasks is assigned, in the form of integrated poster presentation called PosMed, as a part of continuous assessment. In this activity, learners integrate basic science knowledge with medical investigative instruments or tools. Moreover, the students make novel prototypes using scrap materials. They present their research as a poster, which is judged by three invited judges from the Faculty of Medicine and the Faculty of Dentistry. Constructive feedback is provided after each presentation.

**Figure 1:**
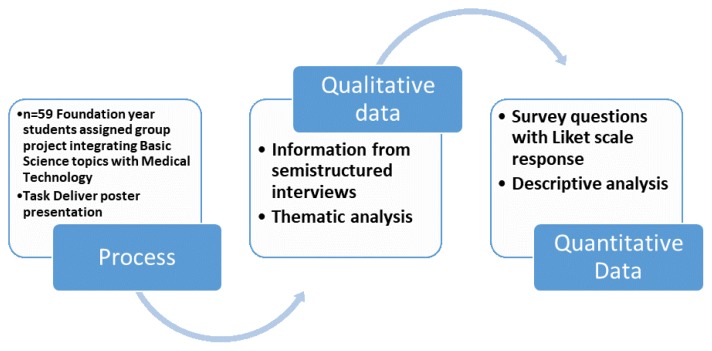
Process adopted for the study

**Figure 2:**
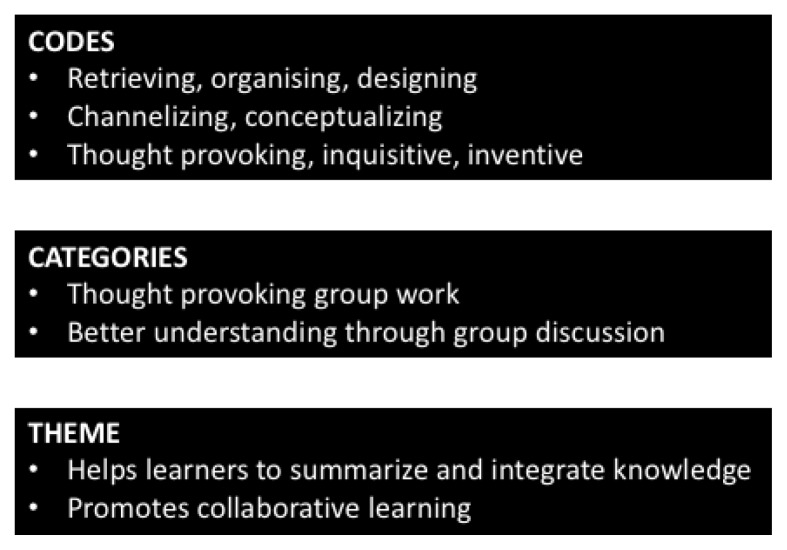
Summary of the thematic analysis used for qualitative data

This interpretative research study used a mixed methods approach for gathering data (Figure 1). A total of 59 foundation year (pre-medical) students participated in this study. The “total population sampling” was used as a variant of purposive sampling in this study. The qualitative data was derived from semi-structured interviews. As the study participants were young population, we used the self-interview technique, in which students were provided with interview guides and interview question printouts that were filled by them in their free time and shared with the investigators. The qualitative analysis of the data was performed using thematic analysis of information from semi-structured interviews (Figure 2). Words that had similar meanings were grouped into emergent major codes, and the major codes were further classified into categories. These categorisations subsequently led to the emergence of major themes. After analysing these themes, to quantify the data, a survey questionnaire was designed on a Likert scale, ranging from strongly disagrees to strongly agree. The questionnaires were vetted locally and internal consistency of questionnaire was determined using pilot testing for calculating Cronbach’s alpha, following which the questions were accordingly adjusted. The study was approved by the institutional research and ethics committee.

## Results

The demography profile of all the participants is depicted in the following Table 1.

### Qualitative Analysis

Students expressed that the PosMed activity honed their skills for team work and collaborative learning.

“During the process I could learn team work ….”

“It gave me opportunity to mix with my friends thereby helping effective team building….”

“I could learn better by sharing my thoughts in a group as well as looking into the discussion of my peers in group….”

The PosMed activity was recognised by many as a process that helped to foster the skills of critical inquiry and active learning.

“This gave me opportunity to reflect, research, raise questions and present in an active manner….”

“The exercise of research proposal writing helped me to synthesise facts and organise information to create a new thinking….”

“It encouraged my critical thinking about my own and other’s actions….”

“By doing inquiry on research topics and developing a proposal I could engage myself in deeper thinking and deeper learning ....”

There were a few concerns shared by some participants especially about resolving conflicts within the group and meeting the deadline in completing the assignment.

“Sometimes we faced difficulty in getting a consensus during multiple opinions in group…”

“I really hate when I find my idea not given credit by the group….”

“Few of my group members were dominant which sometimes prevented myself expressing some of my critical observations….”

“Time constraint was an issue for this assignment….”

### Quantitative Data Analysis

The summary of questionnaire response is given in Table 2.

The students provided positive responses to all statements including enjoyment, self-directed learning, and analysis. The statements that the students marked most highly were as follows: team working (4.27 average), holistic understanding (4.15 average), drawing conclusions (4.14 average), problem solving (4.02 average) and appreciating different viewpoints (4.02 average).

## Discussion

Our results portray that poster presentations are enjoyed by students who see them as a better medium of learning compared with traditional methods. Moreover, poster presentations have the following benefits: enhanced team work skills, improved overall understanding, independent learning, and problem-solving. Moreover, they are beneficial in the memorisation of information too. Writing assignments provide students with the opportunity to reflect, in addition to fostering communicative skills and critical thinking (9). This supports our idea of engaging students in reflective writing after collaborative exercises on proposal writing to foster their critical thinking skills.

The opportunity given to members to experience writing collaboratively not only encourages active learning but also facilitates the experience of working in a team environment (10). This was evident in our study in which most participants agreed on enhancement of their skills of functioning within a team, which can be considered as a major outcome of this intervention. A few participants expressed their concern particularly for resolving certain conflicts that arose within their teams. However, when they realised similar difficulties were shared by others, they eventually felt less isolated in the process. In previously published literature, similar events have been reported (11).

Collaborative engagement within the medical field, both academic and clinical, is commonplace. The Fis programme aims to develop students for the fields that they will be developing in future. The task assigned to students in this project involved an element of independent learning coupled with the integration of knowledge secondary to group activity. Therefore, the concept of collaborative learning was encouraged. The foundation year student who studies basic science via poster presentations can effectively gain some appreciation at both at an individual level and collectively as a team.

The mixed method design was a strength of this study; however, we did not measure critical thinking using any standardised scale, which could be considered as a limitation. Future studies that incorporate such measures could be organised in a same setting for performing an indepth analysis of this topic.

## Conclusion

This study has revealed that poster presentations, when used effectively as an assignment, can facilitate a learner’s critical and reflective thinking and promote active learning. The prior generic guidelines for making the poster were found to be an important step for bringing a systematic scientific approach amongst the learners as well as for integrating basic science and medical knowledge.

## Figures and Tables

**Table 1:** Demography of participants

Variables	Participants (*n* = 59) *n* (%)
Age in years	
< 20	52 (88.13%)
> 20	7 (11.87%)
Gender	
Female	33 (55.93%)
Male	26 (44.07%)
Ethnicity	
Malay	7 (11.87%)
Chinese	17 (28.81%)
Indian	35 (59.32%)
Other	nil

**Table 2:** Analysis of academic benefits through integrated poster presentation

Statements	No & % of students responded Strongly disagree(1), Strongly agree(5)					Avg. Scale from all responses
	1	2	3	4	5	
It helped me memorise facts, ideas or methods from article readings	2 (3.3%)	2 (3.3%)	12 (20.3%)	31 (52.5%)	12 (20.3%)	3.83
It helped me in analysing the basic elements of an idea	0 (0%)	3 (5.1%)	12 (20.3%)	30 (50.8%)	14 (23.7%)	3.93
I can figure out how to use data and ideas to solve problems or complete assignments	0 (0%)	5 (8.4%)	11 (18.6%)	21 (35.5%)	22 (37.2%)	4.02
It helped me to see most issues from multiple points of view	0 (0%)	2 (3.3%)	12 (20.3%)	28 (47.4%)	17 (28.8%)	4.02
It helped me in applying theories or concepts to practical problems or in new situations in the form of designing your own study	2 (3.3%)	2 (3.3%)	9 (15.2%)	31 (52.5%)	15 (25.4%)	3.93
It helped me to pull the ideas together for understanding whole thing	1 (1.6%)	4 (6.6%)	4 6.6%)	26 (44.0%)	24 (40.6%)	4.15
It helped to draw a conclusion from the research	0 (0%)	2 (3.3%)	9 (15.2%)	26 (44.0%)	21 (35.5%)	4.14
It enhanced my team working skills	1 (1.6%)	3 (5.0%)	8 (13.5%)	14 (23.7%	33 (55.9%)	4.27
Tutor comments were not helpful	29 (49.1%)	15 (25.4%)	9 (15.2%)	5 (8.4%)	1 (1.6%)	1.88
Poster presentation is a better medium for learning than traditional tutorials	2 (3.3%)	10 (16.9%)	20 (33.8%)	13 (22%)	14 (23.7%)	3.46
I overall enjoyed the poster presentation assignment	3 (5%)	3 5%)	11 (18.6%)	17 (28.8%)	25 (42.3%)	3.98
